# A very sneaky bug: perspectives of front-line clinicians on whole-genome sequencing for drug-resistant TB

**DOI:** 10.5588/ijtld.22.0335

**Published:** 2022-12-01

**Authors:** B. Memani, J. Furin, H. Cox, A. Reuter

**Affiliations:** 1 Médecins Sans Frontières, Khayelitsha, Cape Town, South Africa; 2 Department of Global Health and Social Medicine, Harvard Medical School, Boston, MA, USA; 3 Division of Medical Microbiology, Institute of Infectious Diseases and Molecular Medicine and Wellcome Centre for Infectious Disease Research, University of Cape Town, Cape Town, South Africa

Dear Editor,

Whole-genome sequencing (WGS) or targeted sequencing of *Mycobacterium tuberculosis* genomic DNA can be used to identify mutations known to confer phenotypic resistance to first- and second-line anti-TB drugs.^1^ WGS-based drug susceptibility testing methods are being rolled out globally and are likely to become increasingly used to guide clinical decision making.^2^,^3^ Little is known, however, about the perceptions and needs of front-line clinicians when it comes to utilising WGS to support the care of people living with rifampicin-resistant TB (RR-TB).^4^.^5^

We therefore undertook an exploratory, qualitative study with physicians working in primary care clinics in Khayelitsha, South Africa, to better understand their perspectives on WGS.^6^ Khayelitsha is home to about 500,000 individuals and is a high HIV and RRTB burden setting, with approximately 200 individuals diagnosed with RR-TB each year.^7^ In the past decade, the Departments of Health from the Province of the Western Cape and the City of Cape Town have worked in partnership with Médecins Sans Frontières (MSF) to provide community-based RR-TB services through 10 primary care clinics. These clinics are staffed by a cadre of 10–15 clinicians involved in the care of people living with RR-TB, and five of them participated in open-ended interviews to ascertain their needs and views on WGS. Data were analysed for theme and content using standard ethnographic approaches.^8^ The study was approved by the Human Research Ethics Committee at the University of Cape Town, Cape Town, South Africa (416/2014), and by the MSF Ethics Review Board, Geneva, Switzerland.

Four of the five clinicians interviewed were women, and all five provided diagnostic and treatment services to people living with drug-resistant TB from the Khayelitsha community. All felt that WGS would provide useful diagnostic information, but they expressed a need for additional training on the methodology. They also highlighted the need for support from laboratory providers and expert clinicians on how to interpret the results. To note, although the clinicians suggested that most RR-TB treatment is standardised in South Africa, a large proportion of people living with RR-TB require some level of individual treatment, and access to more drug resistance information would greatly facilitate more effective, personalised treatment regimens. They noted that the overall care provided to patients takes into account an individual’s clinical needs even in settings where the initial drug regimen design is “standardised”. WGS data could therefore be used to help with adverse event management (i.e., to choose a substitute drug) and to reduce pill burden (i.e., to stop use of a medication that is less likely to be effective and for which there is no other way to obtain drug susceptibility data other than WGS, e.g., data on pyrazinamide). Clinicians were of different opinions, however, about when WGS should be used in addition to the laboratory methods already implemented in South Africa. For example, some felt that all people living with RR-TB should be offered WGS upfront, whereas others felt that WGS should only be used for people who are not doing well, or who have complicated histories or medical issues.

All the clinicians thought that cost would be the biggest barrier to rolling out WGS more widely. Although confident that they could use the test results, and would be well-supported by experts in the laboratory and at the clinical level to deal with more complex interpretations, they were concerned that less experienced providers (or those without access to expert guidance) could struggle with the technology. They felt it would be essential to have clear laboratory reports with the clinical implications of the WGS findings. They also reported they would be comfortable explaining WGS results to patients – especially if they had more training on the results themselves – and thought this would be an important aspect of their work. They felt that they would not need to share all the details of the WGS methods with patients, but rather to let them know that this was additional information on how best to treat their RR-TB.

Providers were specifically asked how they would manage discrepant results from WGS and other methods for assessing drug resistance to TB.^9^ All five clinicians had experience in managing discrepant results with existing tests for TB, and also from their experience of working on HIV. Most said they would reach out to the laboratory to see if there was a reason to account for the discrepancy. They also cited the possibility of a labelling or clinic-based error (i.e., the wrong patient name or number on a sample) as a cause of the discrepancy, and described steps they had taken at their facilities to reduce some errors. Some mentioned that there could also be different populations of mycobacteria in the lungs leading to a discrepant result. All five reported that when discrepant results were not due to labelling or laboratory errors, they assessed the clinical situation of the person living with RR-TB, but they usually treated for the “worst case scenario” based on the most extreme resistance pattern. Some illustrative quotes from participants are presented in the Table.

**Table:** Illustrative comments from participants on themes described in the study

Theme	Key findings	Illustrative quotes
Need for expert laboratory and clinical support	Participants expressed a need for laboratory support to interpret mutations, and for clinical support to understand how mutations would affect regimen selection and management Participants were confident they had such support available to them, but were concerned that others working in different settings might need to have access to support, especially if they were not as familiar with the management of people living with rifampicin-resistant TB	“...So, whole-genome sequencing, at first, you know, it sounds quite intimidating. It sounds as if I’m supposed to know something about genomes, and different mutations, and so on, and interpretations, and that can be quite intimidating, so, and that might put me off. But if I have, from the laboratory, guidance of what the different mutations and things mean, then I think that would be helpful as well.”
Communicating WGS results to patients	Participants reported that they would like additional training to better understand the methods themselves in order to communicate the results to patients Participants expressed the need for tools and for clinic-based support staff to assist with communication, including counsellors, lay health workers and interpreters	“Visual aids will definitely help. An interpreter is very important, because just sitting here, hearing TB, and resistance, and 25 tablets, and heart tests, and it’s overwhelming.”
Communicating discrepant results to patients	Participants reported that they deal with discrepant results with the currently available diagnostic tests, and that they are able to communicate these to patients	“I would have to say something like TB is a very, very sneaky bug that changes the way it looks all the time, so we can’t always recognise it, especially – I will use some sort of analogue that makes sense to somebody that has no background of genome sequencing.”
Perceived barriers to rolling out WGS widely	Participants were concerned about the costs of this additional testing and also the logistical resources needed for it to be used (i.e., electricity)	“Presumably cost and availability and [electricity supply] and all of those kind of things. But I mean the cost will probably be the biggest... But I think if it is possible to individualise treatments, streamline it and make it efficient and effective then that should be the way to go.”
Preferences for standardised or individualised approaches to treatment	Participants reported that even when regimen selection is standardised, they still provide “individualised” care to patients because each person is unique Participants reported that while the “standardised” approach may be more simple and straightforward, there are groups of patients who will need more individualised care (i.e., people with strains that have high levels of resistance, people with histories of previous treatment) Participants were split in terms of offering WGS up front so each patient can have their own treatment plan devised vs. waiting until later or if problems arose	“I can’t really say I lean towards each one, because each patient will be individualised in his own way. Even if his individual plan is to use the general plan but you have to very quickly jump in when you see a patient is veering off the predicted pathway. And then you have to go individual.”

WGS = whole-genome sequencing.

This study had several limitations. First, because only providers working with people from Khayelitsha were included, the results may not reflect those in other programme settings (something that was noted by the providers themselves). Second, we used the term “whole-genome sequencing” to refer to the general modality but did not explore differences in types of genome sequencing that may be easier or more cost-effective. Finally, we did not explore WGS as a replacement for other TB tests that are currently in use; most providers therefore responded on the basis of WGS as an additional service. Future studies should explore these issues in more detail.

Overall, these results show that front-line providers are receptive to the introduction of WGS, but would appreciate having both expert clinical and laboratory support to manage complicated results.^10^,^11^ They reported they would be empowered to communicate the results to people living with RR-TB for whom they were providing services and to make treatment decisions based on these results. However, they had mixed opinions on when WGS should be offered (i.e., upfront for all vs. only for patients who are not doing well or who have complicated histories/clinical scenarios). This last observation likely reflects a general perception of the limited resources that are available in most high RR-TB burden settings.^12^ Given that WGS is rapidly becoming a standard of care in more well-resourced settings, global scale-up of access to WGS should strive to provide the highest standard of care for all individuals with RR-TB. Our results show that front-line providers are ready and willing to implement this approach.
